# Fidelity Characterization of Highly Pathogenic Porcine Reproductive and Respiratory Syndrome Virus and NADC30-like Strain

**DOI:** 10.3390/v16050797

**Published:** 2024-05-16

**Authors:** Xiang Gao, Ting Bian, Peng Gao, Xinna Ge, Yongning Zhang, Jun Han, Xin Guo, Lei Zhou, Hanchun Yang

**Affiliations:** 1 National Key Laboratory of Veterinary Public Health Safety, College of Veterinary Medicine, China Agricultural University, Beijing 100094, China; stu2088@163.com (X.G.);; 2 Key Laboratory of Animal Epidemiology of Ministry of Agriculture and Rural Affairs, College of Veterinary Medicine, China Agricultural University, Beijing 100094, China

**Keywords:** porcine reproductive and respiratory syndrome virus (PRRSV), HP-PRRSV, NADC30-like, fidelity, recombination

## Abstract

The porcine reproductive and respiratory syndrome virus (PRRSV) has significantly impacted the global pork industry for over three decades. Its high mutation rates and frequent recombination greatly intensifies its epidemic and threat. To explore the fidelity characterization of Chinese highly pathogenic PRRSV JXwn06 and the NADC30-like strain CHsx1401, self-recombination and mutation in PAMs, MARC-145 cells, and pigs were assessed. *In vitro*, CHsx1401 displayed a higher frequency of recombination junctions and a greater diversity of junction types than JXwn06. *In vivo*, CHsx1401 exhibited fewer junction types yet maintained a higher junction frequency. Notably, JXwn06 showed more accumulation of mutations. To pinpoint the genomic regions influencing their fidelity, chimeric viruses were constructed, with the exchanged nsp9-10 regions between JXwn06 and CHsx1401. The SJn9n10 strain, which incorporates JXwn06’s nsp9-10 into the CHsx1401 genome, demonstrated reduced sensitivity to nucleotide analogs compared to CHsx1401. Conversely, compared with JXwn06, the JSn9n10 strain showed increased sensitivity to these inhibitors. The swapped nsp9-10 also influences the junction frequency and accumulated mutations as their donor strains. The results indicate a propensity for different types of genetic variations between these two strains and further highlight the nsp9-10 region as a critical determinant of their fidelity.

## 1. Introduction

The porcine reproductive and respiratory syndrome virus (PRRSV) is an economically devastating pathogen that is globally prevalent in most pig-raising countries and regions, such as China [1,2], the United States [3,4], Thailand, Vietnam, Denmark, and many other European countries [5,6,7]. PRRSV infection can cause clinical symptoms, including respiratory distress in pigs of all ages and extensive reproductive failure in sows, such as stillbirths, mummified fetuses, and abortions [8,9,10,11,12,13]. It can also cause immune suppression and establish persistent infections. It is over nearly 30 years since the first identification of PRRSV in the late 1980s [14], and PRRSV prevention and control are still a great challenge for pig farms, even though commercial vaccines have been developed and widely used [15].

PRRSV belongs to the genus *Betaarterivirus* of the family *Arteriviridae* in the order *Nidovirales* [16,17]. There are two genotypes, European (type 1) and North American (type 2) strains, which exhibit significant genetic differences with an nucleotide identity of ~60%, despite having a similar disease phenotype, clinical symptoms, and genomic organization [18,19,20]. Recently, these two species PRRSV-1 (type 1) and PRRSV-2 (type 2) have been classified as *Betaarterivirus suid* 1 and *Betaarterivirus suid* 2, respectively [21,22]. PRRSV is an enveloped virus with a single-stranded, non-segmented, positive-sense RNA genome approximately 15 kb in length, featuring a 5’ cap and 3’ polyadenylated tail [23,24,25]. Its genome comprises at least 10 open reading frames (ORFs), with ORF1a and ORF1b occupying ~80% of the genome and encoding non-structural proteins involved in transcription and replication [26,27,28,29]. The rest of the ORFs encode the structural proteins that consist of the viral particle. Due to its rapid evolution, PRRSV shows significant genetic variability, even within the same genotype, posing challenges for protection and effective vaccine development [30,31,32,33,34]. Genetic variation is a major reason for the new emergence of this pathogen, which can be more difficult to control and sometimes causes more severe disease in pigs. A multitude of new strains has sustained the global epidemic of this disease, supported by high rates of mutation and recombination [35,36,37].

Viral replication fidelity refers to the accuracy with which a virus copies its genetic material during replication [38]. This accuracy can vary depending on the type of virus [39,40]. It has been a well-accepted concept that RNA viruses have lower fidelity compared with DNA viruses, characterized by rapid evolution and extensive genetic diversity, endowing them with substantial adaptive potential [41]. However, this adaptability comes at a cost, as low-fidelity replication imposes a limit on genome size, typically not exceeding ~15 kb [42,43,44]. Low fidelity in replication is believed to be a primary driver of RNA viruses’ rapid evolution and adaptation to new host species and environmental pressures, leading to relatively high mutation and recombination rates per replication cycle [45,46,47]. As an RNA virus, PRRSV has a notably high mutation rate due to the lack of RNA proofreading during replication [30]. Previous studies have shown that PRRSV evolves through random mutations and intragenic recombination during infections, contributing to the emergence of new strains and their associated virulence [48,49,50,51,52]. The increased genomic variation leads to the phenotypic diversity of different PRRSV strains.

In China, highly pathogenic PRRSV (HP-PRRSV) was previously the predominant strain in the field during the years of 2006–2013 and was then gradually replaced by NADC30-like strains, and there is currently a higher ratio of recombinant strains using them as parental viruses [37,53]. In addition, more nsp2 deletion patterns were identified in these two kinds of strains [37,54,55,56]. While NADC30-like PRRSV is considered to be of relatively milder virulence compared to HP-PRRSV, it has been associated with clinical respiratory symptoms in all-age pigs and later-term abortion, with a rate of 30–40% in sows [57,58,59]. Despite being less aggressive than HP-PRRSV, the NADC30-like strain and its recombinant strains often show persistent infection with a longer course of disease [60,61,62]. Moreover, current commercial vaccines can only provide limited cross-protection against NADC30-like PRRSV, so it still poses a serious health risk to pig herd [63,64,65]. However, the replication fidelity of these two kinds of strains is less reported. In this study, the fidelity characterization of HP-PRRSV and NADC30-like strains was investigated in infected porcine alveolar macrophages (PAMs), MARC-145 cells, and pigs. In addition, the fidelity characterization of two chimeric viruses with swapped nsp9 and nsp10 between HP-PRRSV and the NADC30-like virus was also investigated. The findings will provide a data basis for understanding the evolutionary patterns and epidemiological characteristics of these different PRRSV strains.

## 2. Materials and Methods

### 2.1. Ethical Statements

The animal experiments in this study were carried out according to the Chinese Regulations of Laboratory Animals: The Guidelines for the Care of Laboratory Animals (Ministry of Science and Technology of the People’s Republic of China) and Laboratory Animal Requirements of Environment and Housing Facilities (National Laboratory Animal Standardization Technical Committee). The protocol for primary PAM preparation was approved by the Laboratory Animal Ethical Committee of CAU, with the approval No. AW81801202-2-1.

### 2.2. Virus and Cell Culture

The HP-PRRSV representative strain JXwn06 (GenBank accession number: EF641008) and NADC30-like virus CHsx1401 (GenBank accession number: KP861625.1) were used in this study, whose sequences, pathogenicity, and immune response in the infected animals are well documented in previous studies [66,67]. Primary PAMs were obtained from the lungs of one-month-old pigs. The lung was removed without breakage, and lung lavage was performed with 4% RPMI 1640 (Gibco, Grand Island, NY, USA) in PBS. The lavage solution was centrifuged, and the cells were counted. The PAMs were maintained in RPMI 1640 medium (Gibco) supplemented with 10% fetal bovine serum (FBS Gibco) and a combination of penicillin (50 U/mL) and streptomycin (50 mg/mL) in a 37 °C incubator with a 5% CO_2_ atmosphere. MARC-145 cells, the derivative of the African monkey kidney MA-104 cell line, were cultured in Dulbecco’s modified Eagle’s medium (DMEM) (Gibco) supplemented with 10% fetal bovine serum (Gibco), penicillin (50 U/mL), and streptomycin (50 μg/mL). The culture was maintained at 37 °C in the incubator with a 5% CO_2_ atmosphere.

### 2.3. Sera Samples from PRRSV-Inoculated Pigs

The sera samples utilized in this study were obtained from our laboratory’s reserves. One-month-old specific-pathogen-free (SPF) Large White pigs, purchased from the Beijing SPF Swine Breeding Management Center, were intranasally inoculated with 2 × 10^5^ TCID_50_/mL, JXwn06, or CHsx1401, respectively, and the experiment lasted for 21 days. At the end of the experiment, all surviving animals were humanely euthanized using exsanguination under anesthesia and were disposed of with care. The sera samples collected at 0, 3, 7, 14, and 21 days post-inoculation (dpi) were tittered, and 3 sera samples from each group collected at 7 dpi at the peak of viremia were submitted for NGS.

### 2.4. Preparation of Full Viral Genomes for Deep Sequencing

The PAMs or MARC-145 cells were infected with PRRSV JXwn06 or CHsx1401 at an MOI of 0.01 for 24 h, and then the samples were harvested and put into a −80 °C freezer. Blood samples were collected from the pigs inoculated with PRRSV JXwn06 or CHsx1401, respectively, at 7 days post-inoculation, and the sera were obtained at room temperature. Viral RNA was extracted from the harvested cells or sera using the TRIzol reagent (Invitrogen, Waltham, MA, USA), including the viral genome, subgenomic RNA, and defective viral genome (DVG), followed by reverse transcription using a FastKing RT kit (Tiangen, Beijing, China) with 14 primers, which covered the whole genome (Tables S1 and S2), and the cDNA was a synthesized complementary strand prepared for Next-Generation Sequencing (NGS).

### 2.5. Short-Read Illumina Sequencing of Viral cDNA

NGS libraries were generated using 2 μg of cDNA of each sample. The cDNA samples were sent to the sequencing company Baihuiyineng for library preparation, and 2 × 150 nucleotide paired-end sequencing was subsequently performed (Illumina, San Diego, CA, USA).

### 2.6. Illumina RNA-Seq Processing and Alignment

*Trimmo-matic* was employed for processing raw reads to eliminate Illumina adapters, applying default settings (command line: *trimmomatic PE sample.raw.R1.fq.gz sample.raw.R2.fq.gz sample_1_paired.fq.gz sample_1_unpaired.fq.gz sample_2_paired.fq.gz sample_2_unpaired.fq.gz ILLUMINACLIP: TruSeq3-PE.fa:2:30:10 LEADING:3 TRAILING:3 SLIDINGWINDOW:4:15 MINLEN:36*] [68]. Reads under 36 bp in length were discarded, and ends of reads with low-quality bases (Q scores < 30) were trimmed. Alignment of the fastq files to their respective sequences was conducted using the *ViReMa* Python2 script (Viral Recombination Mapper, version 0.29) [69], following the command line *python ViReMa.py sample.fq.gz reference_index input.fastq sample.sam -BED –MicroInDel_Length 5 –Output_Dir sample_virema*. Subsequently, the sequence alignment map (SAM) file was processed using *samtools* to ascertain the nucleotide depth at each position within a sorted binary alignment map (BAM) file, using the command line *samtools depth -a -m 0 sample_virema.sort.bam > sample_virema.coverage.txt* [68].

### 2.7. Recombination Junction and Nucleotide Mutation Analysis

In determining the frequency of genomic junctions, a comparison was made between the nucleotides implicated in these junctions and the total mapped nucleotides. The number of nucleotides at these junction sites, as identified by *ViReMa* in the BED files, was aggregated for quantification. This total mapped nucleotide count was assessed using the *sample_virema.coverage.txt* file described above. Due to the difference in replication speed between different strains, we plotted junction patterns at approximately identical coverage, using *seqkit* to determine the coverage.

The mutation rate of each virus was calculated by comparing the number of mutations to the aggregate number of nucleotides at each position in the genome. Mutations were selectively excluded from counting if their relative proportion was lower than 0.001. The total nucleotide count was determined by aggregating the depth of nucleotides at each position throughout the genome, as indicated in the *samtools*-generated coverage files. Mutations were considered significant and recorded if their proportions were above 1%.

### 2.8. qRT-PCR Analysis

For qRT-PCR analysis, total RNA from the supernatant, intracellular, and cell lysate was extracted by the TRIzol reagent (Invitrogen) and purified. The FastKing One Step Probe RT-qPCR kit (TIANGEN) and commercial PRRSV detection qPCR kit from Yishengbao were used to quantify the viral RNA, with each 50 μL reaction containing 5 μL of RNA, 10 μM of each primer, and a 200 pmol probe. The reverse transcription qPCR was performed at 50 °C for 10 min and 95 °C for 3 min, followed by 40 cycles at 95 °C for 15 s and 60 °C for 30 s in the Bio-Rad CFX96 Touch qPCR System. The standard curve (y = −3.9004x + 55.20, R^2^ = 1.00) was generated using a plasmid with the target sequence.

### 2.9. Cloning, Recovery, and Verification of Chimeric Virus

The CMV-promoter-driven PRRSV infectious clones of JXwn06 and CHsx1401 were modified to generate the chimeric viruses with swapped nsp9 and nsp10 (nsp9-10)-coding regions. These chimeric clones were produced by incorporating the respective gene segment into individual DNA fragments of the PRRSV infectious clone by fusion PCR [70]. As per the manufacturer’s guidelines, the sequenced full-length plasmids were transfected into MARC-145 cells via Lipofectamine™ LTX Reagent (Thermo Fisher, Waltham, MA, USA). The MARC-145 cells in six-well plates were frozen at 5 days post-transfection, and subsequently, the lysate was passaged in MARC-145 cells for three generations. In the third passage, the viral genomes were extracted for additional verification by sequencing.

### 2.10. Immunofluorescence Assay

MARC-145 cells in six-well plates, infected with the virus for 48 h, were fixed using absolute ethanol for 30 min and subsequently washed twice with phosphate-buffered saline (PBS). The cells were then incubated at 37 °C for 1 h with a monoclonal antibody (MAb) targeting the PRRSV N protein, produced by our lab at China Agricultural University, as previously described by Can Kong [71]. Following being washed twice with PBS, the cells were incubated for an additional hour with the goat anti-mouse secondary antibody conjugated with Fluorescein Isothiocyanate Isomer I (FITC). Nuclear DNA was stained with 4’,6-diamidino-2-phenylindole (DAPI) for 10 min, followed by a triple wash with PBS. Imaging was taken using the Nikon Eclipse Ti-U Inverted Fluorescence Microscope, with images merged using NIS-Elements D software (Nikon, Tokyo, Japan).

### 2.11. Nucleotide Analog Inhibition Assays

Ribavirin (Sigma, Darmstadt, Germany) was prepared in a 200 mM aqueous solution. Similarly, 5-FU (Sigma) was prepared in a 200 mM solution using dimethyl sulfoxide (DMSO), and 5-AZC (Sigma) was prepared in a 100 mM aqueous solution. MARC-145 cells were subjected to a one-hour pretreatment with DMEM with or without varying concentrations of ribavirin, 5-FU, or 5-AZC, respectively. Post-treatment, the cells were washed twice with PBS before inoculating with PRRSV at an MOI of 0.01 and incubating for one hour at 37 °C. After the inoculation phase, the inoculum was removed, and the cells were further cultured in the medium with or without the nucleoside analog. The experiment proceeded for 24 h, after which the cells were frozen. Virus titers were then evaluated using the 50% tissue culture infective dose (TCID_50_) assay [72].

### 2.12. Statistical Analysis

GraphPad (La Jolla, CA, USA) Prism 8 software was used for applying the statistical analysis. Two-tailed Student’s *t*-test was used to test the differences between the two groups, and the differences among multiple groups were analyzed by two-tailed unpaired Student’s *t*-test or one-way ANOVA. Data are shown as the mean ± SD of the biological triplicates. Details of the statistical analysis for each experiment are indicated in the relevant figure legends. For all statistical tests, *p*-values < 0.05 were considered statistically significant.

## 3. Results

### 3.1. Replication Fidelity Characterization of HP-PRRSV and NADC30-like Virus in PAMs

**Figure 1:**
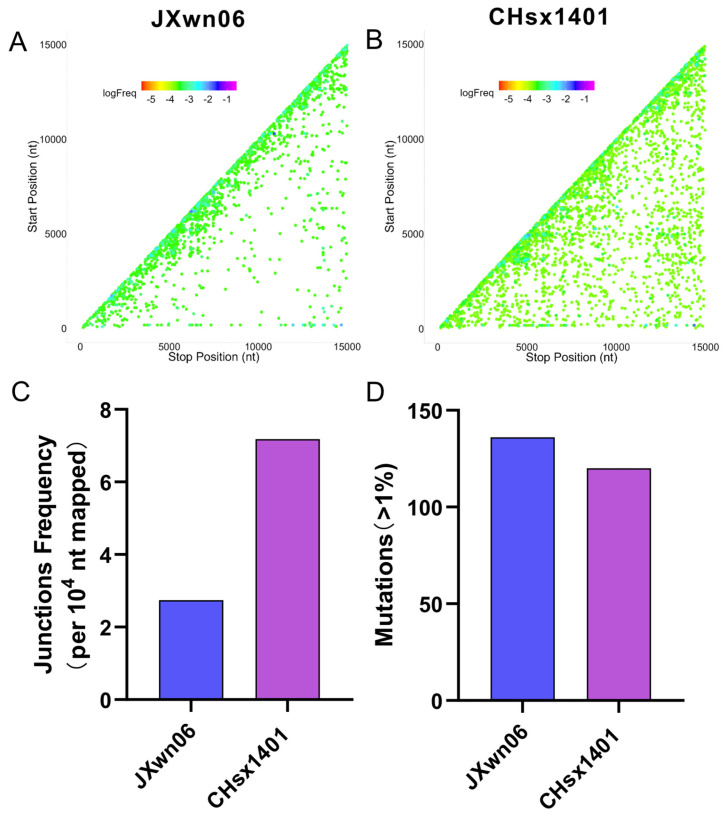
Recombination and mutation analysis of HP-PRRSV and NADC30-like virus. (**A**,**B**) The scatter plots are generated by junctions of HP-PRRSV strain JXwn06 (**A**) and NADC30-like strain CHsx1401 (**B**) mapping to a specific position (5′ junction site, Start position; 3′ junction site, Stop position). Each spot is colored according to its frequency in the population of all junctions, and the colors transition from a cool-toned to a warm-toned spectrum, corresponding to the frequency in all junctions from the highest to the lowest. (**C**) The junction frequency was statistically analyzed with the overall nucleotides mapped to the viral RNA, divided by the nucleotide involved in junctions detected by *ViReMa*. For both JXwn06 and CHsx1401, the junction frequency was quantified by calculating the number of junctions per 10,000 mapped nucleotides. (**D**) The accumulated mutation number of JXwn06 and CHsx1401 in the library, with a proportion exceeding 1%, is shown in the histogram.

To characterize the replication fidelity of HP-PRRSV and the NADC30-like virus, the recombination junction and nucleotide mutation of the representative strains JXwn06 (HP-PRRSV) and CHsx1401 (the NADC30-like virus) were quantified by NGS. The high sequence depth and low error rate of NGS greatly facilitated in identifying and quantifying unique junctions and mutations, detecting both those in high and low abundance. Reads were aligned to respective viral genomes using *ViReMa* (Virus Recombination Mapper) [69]. *ViReMa* detected both recombined and non-recombined reads in the library and output the total number of mapped nucleotides and all detected recombinant junctions. To define the pattern of self-recombination junctions of JXwn06 and CHsx1401, respectively, the forward (5′->3′) recombination junctions were mapped according to their position, comparing nearly identical read coverages for JXwn06 (1870) and CHsx1401 (1703) (Figure 1A,B). Both JXwn06 and CHsx1401 viruses had local deletions across the genome. In addition, CHsx1401 had much more low-frequency junctions with deletion over 4000 nt across the whole genome. When statistically analyzing the junction frequency of each virus, CHsx1401 had approximately 3-fold higher junction frequency than that of JXwn06 (Figure 1C), while JXwn06 had slightly more accumulative mutation than that of CHsx1401 (Figure 1D). Overall, these data demonstrate that extensive RNA self-recombination of JXwn06 and CHsx1401 was generated diversely during replication with similar high-abundance clusters.

### 3.2. The NADC30-like Virus Generated a Higher Proportion of Non-Infectious Genome Compared to HP-PRRSV

**Figure 2:**
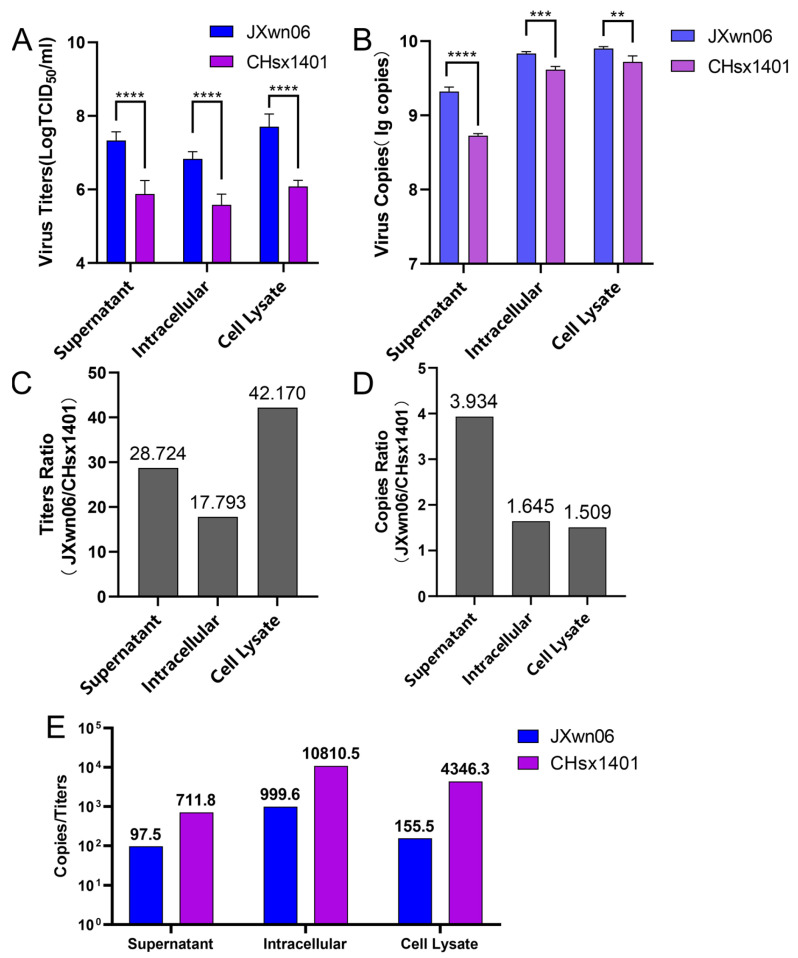
Viral titers and viral RNA ratio analysis of HP-PRRSV and NADC30-like strain. (**A**) JXwn06 and CHsx1401 infected MARC-145 cells at MOI of 0.01, and the virus was harvested at 48 h post-infection. The virus in the supernatant, intracellular, and cell lysate was quantified using 50% tissue culture infectious dose (TCID_50_) assay, respectively. Each sample was tested in three independent experiments. (**B**) Viral RNA was also extracted from the samples collected above, and then the copies of the ORF6 gene in JXwn06 and CHsx1401 were detected by one-step real-time PCR. Three independent experiments were repeated. The viral titer (**C**) and viral RNA(**D**) ratio of JXwn06 and CHsx1401 in the supernatant, intracellular, and cell lysate were calculated with the mean value of each virus, respectively. (**E**) The ratios of copies/titers of JXwn06 and CHsx1401 in supernatant, intracellular, and cell lysate were compared. All columns of JXwn06 are represented in blue, and columns of CHsx1401 are represented in purple. (** *p* < 0.01, *** *p* < 0.001, **** *p* < 0.001).

To generally compare the ratio of non-infectious genome generated during the viral replication of these two strains, JXwn06 and CHsx1401 infected MARC-145 cells at an MOI of 0.01 for 48 h. Then, the quantity of viral titer and viral RNA in the supernatant, intracellular, and cell lysate of infected MARC-145 cells was determined. The viral titers of JXwn06 in the supernatant, intracellular, and cell lysate were all higher than that of CHsx1401 at 48 h post-infection (Figure 2A). For the viral RNA copies assessment, JXwn06 also had more copies of the ORF6 gene than that of CHsx1401 in the supernatant, intracellular, and cell lysate (Figure 2B). To analyze the replication difference between JXwn06 and CHsx1401, we compared the viral titer and viral RNA copies between the two strains. The largest viral titer ratio of JXwn06/CHsx1401 was in the comparison of cell lysate at about 42.8-fold (Figure 2C). The viral titer ratio in the supernatant and intracellular exhibited a 28.7-fold and 17.8-fold difference, respectively. However, the largest viral RNA copies ratio of JXwn06/CHsx1401 was only about 3.9-fold in the comparison of the supernatant (Figure 2D). In addition, the ratio of cell lysate and intracellular was approximately 1.5-fold and 1.6-fold. It was found that the ratio of the viral titer of JXwn06/CHsx1401 was higher than the ratio of the viral genome copies. To better illustrate the diversity of infectious genomics between JXwn06 and CHsx1401, the copies/titer ratio of these two viruses was further compared. CHsx1401 had a higher ratio value than JXwn06 in all three items (Figure 2E), indicating that CHsx1401 might generate a more non-infectious genome compared to JXwn06.

### 3.3. The NADC30-like Strain Has a Higher Frequency of Recombination Compared to HP-PRRSV In Vivo

**Figure 3:**
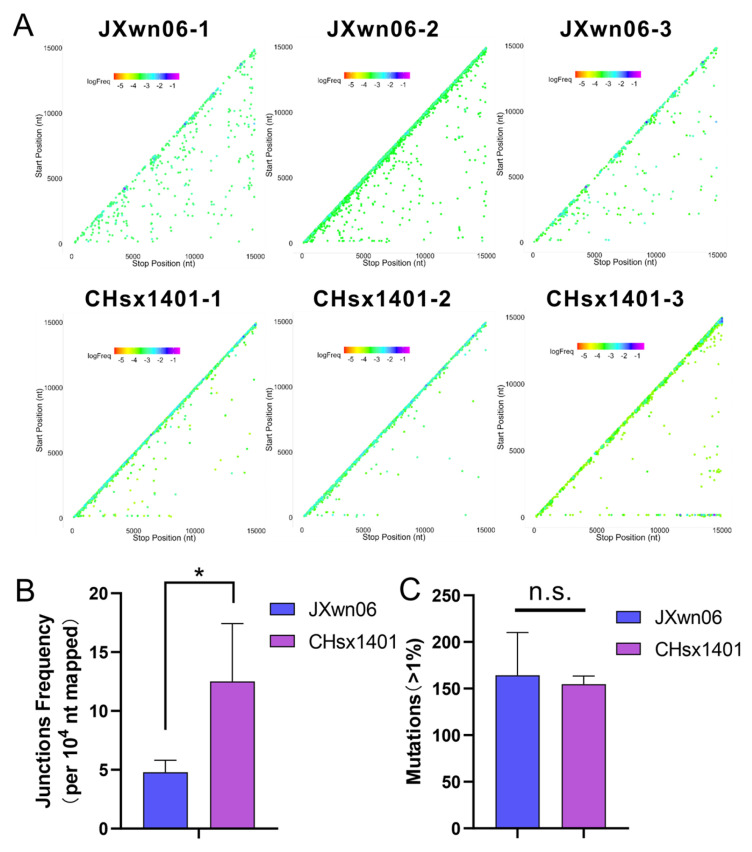
Recombination and mutations analysis of HP-PRRSV and NADC30-like virus *in vivo*. (**A**) The viral RNA in serum samples from JXwn06- or CHsx1401-inoculated pigs was sequenced by NGS and aligned to the respective genome using *ViReMa*. The junction distribution of JXwn06 and CHsx1401 *in vivo* is shown in a scatter plot. The spots are colored according to the frequency in all junctions in each library. Each junction is marked with the Start position (5′ junction site) and the Stop position (3′ junction site). (**B**) Junction frequency was calculated by counting the number of nucleotides in junctions detected with *ViReMa* and comparing it to total mapped nucleotides. (**C**) The accumulated mutation number of JXwn06 and CHsx1401 *in vivo*, with a proportion exceeding 1%, is shown in the histogram. (n.s.: no significance, * *p* < 0.05).

Previous studies have shown that there have been many recombinant viruses in Chinese fields since 2013, predominantly characterized by recombination among Lineages 1, 3, 5, and 8 [37]. Notably, recombination between the NADC30-like virus (Lineage 1) and HP-PRRSV (Lineage 8) represents a significant portion of these cases. To test the replication characterization of HP-PRRSV and the NADC30-like virus *in vivo*, serum samples from each of the three pigs infected with JXwn06 or CHsx1401, collected at 7 days post-inoculation, were submitted to viral RNA extraction and transcription for NGS. Based on the same analysis methods *in vitro*, the patterns of the detected recombination junctions in the JXwn06 group showed more long-segment deletions than CHsx1401 *in vivo*, at a coverage of around 800–900 (Figure 3A). In contrast, CHsx1401 generated more self-recombination (junction frequency) than JXwn06 *in vivo* (Figure 3B). The mean number of accumulative mutations was higher in the JXwn06 group, although there is not a statistically significant difference between JXwn06 and CHsx1401 (Figure 3C).

### 3.4. The Modification of nsp9-10 Can Impact PRRSV’s Sensitivity to Nucleoside Analog

**Figure 4:**
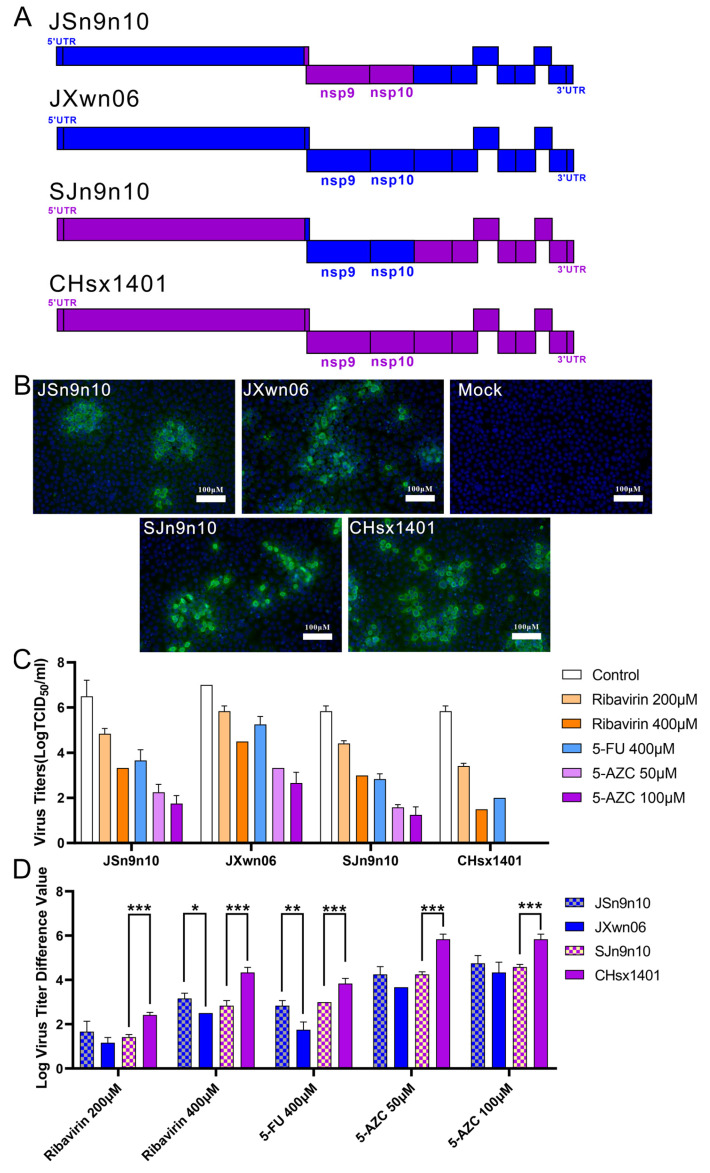
The nucleoside analog resistance of chimeric viruses compared to their parental strains. (**A**) The schematic diagram of the construction of chimeric viruses JSn9n10 and SJn9n10. (**B**) Identification of the rescued virus in MARC-145 cells by immunofluorescence assay at 48 h post-infection. (**C**) Assessing the nucleoside analog resistance of chimeric viruses JSn9n10 and SJn9n10 and their parental strains against ribavirin, 5-FU, and 5-AZC. MARC-145 cells underwent infection at an MOI of 0.01 with a period lasting 24 h of infection. Virus titers were determined using TCID_50_ assay, and the data reflect the outcomes of three independent experiments. (**D**) The virus titer difference between with and without nucleoside analog treatment in 24 h is shown in the histogram. (* *p* < 0.05, ** *p* < 0.01, *** *p* < 0.001).

According to previous reports, fidelity is mainly determined by the RdRP domain [73,74], the central part of the RTC, which is contained in nsp9 in PRRSV. It has been also found that the viral helicase operates in combination with the polymerase to further alter replication complex fidelity [75]. Meanwhile, the nsp9- and nsp10-coding regions together are found to be closely related to replication efficiency both *in vitro* and *in vivo* [76]. To further evaluate if these two genes impact replication fidelity, two chimeric viruses were constructed with swapped nsp9- and nsp10-coding regions between JXwn06 and CHsx1401. The chimeric strain JSn9n10 was generated by substituting the nsp9-10-coding region of CHsx1401 into the backbone virus JXwn06; in contrast, the virus SJn9n10 uses CHsx1401 as the backbone virus and replaces the nsp9-10 region from JXwn06 (Figure 4A). The chimeric and parental strains were recovered in MARC-145 cells and passaged three generations, which was further confirmed by sequencing and an immunofluorescence assay (Figure 4B).

Numerous studies have utilized mutagenic nucleoside analogs, such as ribavirin, 5-FU, and 5-AZC, to evaluate viral fidelity [77,78,79,80]. These antiviral mutagens increase the mutation rate of RNA viruses during replication beyond the tolerable error threshold, maintaining a lethal mutagenic effect that ultimately leads to the extinction of the viral infection. To investigate the fidelity of chimeric strains, compared with their parental strains, their resistance to the nucleotide analog of each strain was tested. Virus-infected MARC-145 cells at an MOI of 0.01 with or without ribavirin, 5-FU, and 5-AZC, and the infected MARC-145 cells were harvested at 24 h post-infection, and the lysate was tittered with a TCID_50_ assay. The viral titers and corresponding concentrations of each nucleotide analog are shown in Figure 4C. The titer difference between with and without nucleotide analog treatment is shown to provide insights into the effectiveness of the nucleotide analog in inhibiting viral replication (Figure 4D). When comparing the reduced titers via the nucleotide analog treatment, JXwn06 shows less difference than CHsx1401. In addition, the JSn9n10 strain shows lightly increased sensitivity to ribavirin and 5-FU when compared with the parental strain JXwn06, while the SJn9n10 strain shows significantly increased resistance to all three kinds of nucleoside analog when compared with its parental strain CHsx1401. The results above indicate that the nsp9-10 region might relate to the fidelity difference between HP-PRRSV and the NADC30-like virus.

### 3.5. Swapped nsp9-10 Related to the Fidelity Difference between JXwn06 and CHsx1401

**Figure 5:**
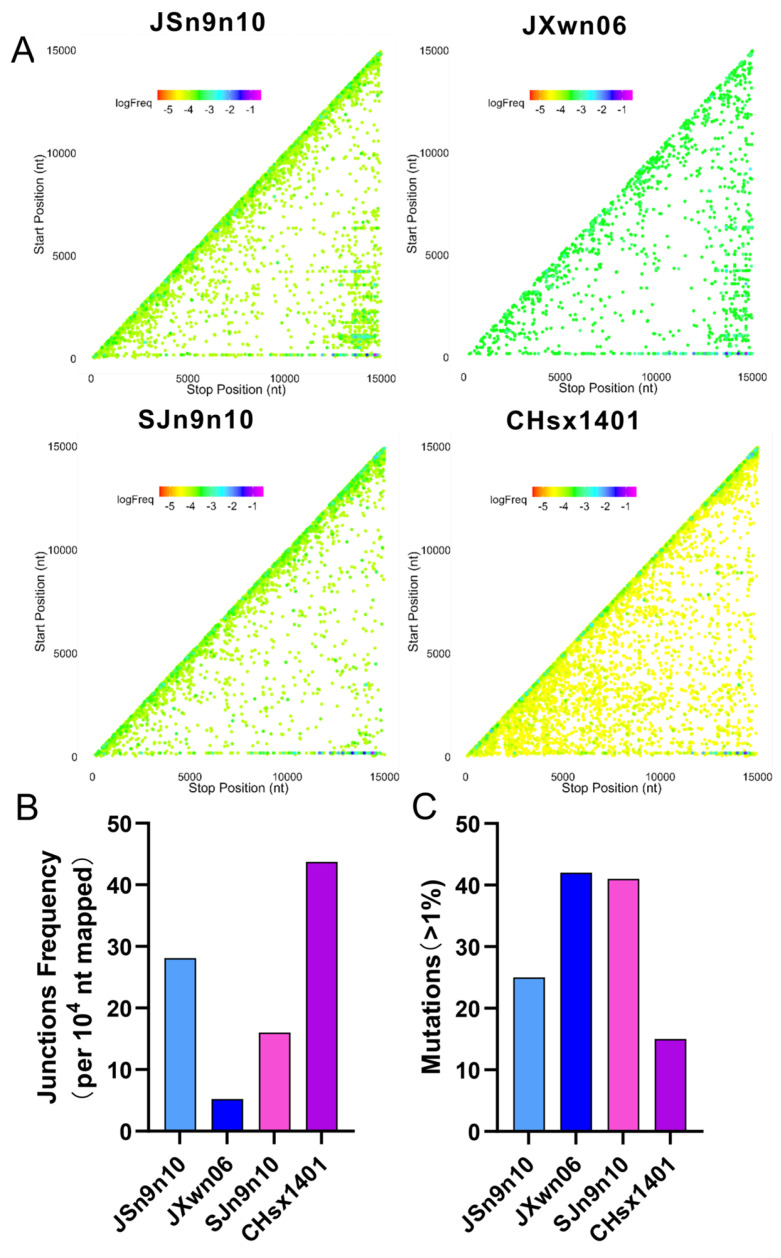
Recombination and mutation analyses of chimeric virus through NGS. (**A**) Recombinant junctions are mapped to the viral genome according to the site of each junction (5′ junction site, Start position; 3′ junction site, Stop position). The spot is colored depending on its proportion in all junctions. Warm-toned spots represent lower proportions, and cool-toned spots represent higher proportions. (**B**) The determination of junction frequency entailed normalizing the count of nucleotides at junction sites, identified by *ViReMa*, to the total nucleotides mapped onto viral RNA. The junction frequencies of both chimeric and parental strains are presented as the number of junctions per 10,000 mapped nucleotides. (**C**) The accumulated number of mutations in each sample, with a proportion exceeding 1%, is shown in the histogram.

Further, we sought to identify recombination patterns and quantify recombination frequency in two chimeric viruses and their parental strains. MARC-145 cells were infected with the respective strains at an MOI of 0.01, and total RNA was extracted at 48 h post-infection. The nucleic acid sample was prepared, sequenced, and analyzed, as described above. The recombination was mapped according to their genomic position, comparing nearly identical read coverages for JXwn06 (1368), CHsx1401 (1398), JSn9n10 (1288), and SJn9n10 (1204) after being extracted by *seqkit* (Figure 5A). According to the statistics analysis of *ViReMa*, the self-recombination patterns of JSn9n10 were more complicated than those of JXwn06. In addition, SJn9n10 had fewer self-recombination junction types than CHsx1401. Meantime, JSn9n10 generated more long-segment deletions compared to JXwn06, while SJn9n10 generated fewer long-segment deletions compared to CHsx1401. In terms of the junction frequency statistics, JSn9n10 generated more recombination than JXwn06, while SJn9n10 generated less recombination than CHsx1401 (Figure 5B), indicating the nsp9-10 region is related to the frequency of recombination in PRRSV. The JSn9n10 strain, with the nsp9-10 from CHsx1401, generated fewer accumulative mutations compared to JXwn06, whereas the SJn9n10 strain, with the nsp9-10 from JXwn06, had more accumulative mutations compared to CHsx1401 (Figure 5C). Overall, the recombination and mutation analysis by NGS further confirms that the nsp9-10 shows a determinant effect in regulating the fidelity of PRRSV.

## 4. Discussion

Recombination, significant in the evolution of many RNA viruses, is primarily facilitated by a mechanism known as “copy-choice” recombination [81]. In this process, the RNA-dependent RNA polymerase (RdRP) mediating viral replication shifts from one RNA molecule (donor template) to another (acceptor template) to produce an RNA molecule of mixed ancestry [82,83,84]. Utilizing NGS and *ViReMa* analysis [69], we monitored self-recombination in HP-PRRSV and the NADC30-like virus during replication in PAMs and mapped the distribution of recombination in the viral genome. CHsx1401 had a higher frequency of junctions and more diversity of junction types than JXwn06, including the generation of large fragment deletions. Comparing the replication titers and gene copies in infected MARC-145 cells confirmed that CHsx1401 might produce more non-infectious genomes. Interestingly, the trends of junction patterns in these two strains are not consistent in the tests *in vivo* and *in vitro*. Whether this difference is due to the immune response within the pig or other reasons still needs further determination.

The self-recombination frequency in RNA viruses is closely associated with the fidelity of the viral replicase, which significantly influences the likelihood of recombination events during co-infection with other strains. A lower fidelity or higher error rate can lead to more frequent template switching during replication, a process that is central to the “copy choice” model of RNA recombination. This model posits that the RdRP may switch from one RNA template to another while synthesizing the new strand, thereby generating a recombinant molecule with mixed genetic ancestry [85]. However, recombination frequencies in RNA viruses are multifactorial, affected not only by replicase fidelity but also by factors such as the degree of sequence identity between templates, the secondary structure of RNA, the multiplicity of infection (MOI), and the specific biology of the virus–host interaction. Additionally, the evolutionary pressures acting on the viral genome organization and life cycle of the virus can shape the diversity of recombination rates observed among different RNA viruses. Thus, while replicase fidelity is a key determinant of self-recombination, it is part of a complex interplay of factors that ultimately dictate the frequency and significance of recombination in RNA virus populations.

Due to the lack of 3′ to 5′ exonuclease proofreading, RNA viruses have a high mutation rate [86]. It is posited that RNA viruses have an error threshold, with any increase in mutation rates potentially leading to the collapse of viral populations [87]. In both *in vitro* and *in vivo* conditions, JXwn06 accumulates more mutations than CHsx1401. Considering the distinctions in recombination, it is possibly indicating a preference for different types of variations between the two strains. Exposing RNA viruses to mutagens, commonly ribavirin, 5-FU, and 5-AZC, has empirically tested pushing viral populations beyond this error threshold. As concentrations of the nucleoside analog increase, so does the virus mutation frequency until the viral population succumbs to extinction [77,78,88]. Introducing low-fidelity mutations into the RNA virus genome results in a reduction in titer with or without nucleoside analog treatment [89,90]. We constructed chimeric viruses with modified nsp9-10 to identify fidelity determinants by comparing nucleoside analog inhibition to parental strains. Recombination and mutations in chimeric strains with modified nsp9-10 showed significant differences to parental strains, highlighting nsp9-10 as a determinant region regulating PRRSV fidelity.

The RNA-dependent RNA polymerase is the core for RNA virus replication, serving as a key regulator of nucleotide selection and fidelity [73,74]. Despite conserved motifs [91,92], the RdRP shares limited amino acid sequence identity across RNA viruses, while RdRp structures commonly resemble a “cupped right hand” structure with finger, palm, and thumb domains [92,93]. The palm domain contains the active site, and the thumb domain contacts synthesized RNA. The finger domains create a pathway for the template RNA and ribonucleotide triphosphates (rNTPs) to enter [94,95,96]. Viral genes encoding the RdRp often incorporate additional domains for various functions, such as methyltransferase and endonuclease activities [97,98,99]. PRRSV nsp9 contains the RdRp domain and a putative nucleotidyltransferase at the N-terminus, whose role remains to be demonstrated. Studies on poliovirus and other RNA viruses have identified specific amino acid residues within the RdRp that are crucial for fidelity, which can be modulated to influence viral evolution and stability [91,100,101,102]. The PV mutant with 3D polymerase G64S high-fidelity mutation is a strain with increased fidelity obtained through ribavirin passage screening [103]. Although no significant replication differences were observed *in vitro*, its pathogenicity in mice was much lower than that of the wild-type strain, with a 50% lethal dose (LD_50_) 300 times higher than the wild-type strain, and it was unable to infect the central nervous system or be shed through feces [104,105,106]. Another experiment showed that mice inoculated with the PV mutant with the 3D polymerase T362I low-fidelity mutation had a higher survival rate compared to the wild-type strain [90]. Additionally, the CVB3 low-fidelity mutant exhibited reduced titers in several target organs of the mouse model and was unable to establish a persistent infection [91]. This suggests that mutations can also alter the pathogenicity and transmission capabilities of the virus. However, inconsistencies in the observed phenotypes suggest viral fidelity is more complex than previously thought. The exact region or even sites that determine PRRSV fidelity are still under study in our current project.

## 5. Conclusions

The replication fidelity differences between HP-PRRSV and the NADC30-like virus were analyzed in PAMs, MARC-145 cells, and pigs by comparing their self-recombination and accumulative mutations. The data reveal the NADC30-like strain CHsx1401 generated more recombination, while JXwn06 generated more accumulative mutations, suggesting a propensity for different types of genetic variations between these strains. In addition, the fidelity evaluation of chimeric viruses with swapped nsp9-10 segments between JXwn06 and CHsx1401 further indicated the nsp9-10 region as a critical determinant of their fidelity. The study provides insights into the genetic underpinnings of PRRSV evolution and epidemic characterization.

## Data Availability

The PRRSV strains mentioned in this study are available in GenBank with the accession numbers: JXwn06 (EF641008) and CHsx1401 (KP861625).

## Supplementary Materials

The following supporting information can be downloaded at: https://www.mdpi.com/article/10.3390/v16050797/s1, Table S1: Primers sequence for JXwn06 reverse transcription; Table S2: Primers sequence for CHsx1401 reverse transcription.
