# Supplementary material for: Fidelity Characterization of Highly Pathogenic Porcine Reproductive and Respiratory Syndrome Virus and NADC30-like Strain
Source: Viruses. 2024 May 16;16(5):797. doi: 10.3390/v16050797 (PMC11125636; doi:10.3390/v16050797)
Supplement: Supplementary file 1 [file viruses-16-00797-s001.zip › viruses-3003091-supplementary.pdf]

Supplementary Materials

Table S1. Primers sequence for JXwn06 reverse transcription.

Name	Sequence	Position
JX1R	TACTCTTTCAGGAAGGGTGG	1575-1556
JX2R	AGGTTGTTCGGTTGTCTGATT	2253-2233
JX3R	CGATGATGGCTTGAGCTGAGTAT	3178-3156
JX4R	GGCGATCTCATTAGGAGCAGTT	4329-4308
JX5R	ACGGTGTTCAGTGAGGGCTTT	5564-5544
JX6R	AAGGAAATCCAAGTCCTCGTC	6750-6730
JX7R	CCAAAGCGTGCCATCAATCCC	7922-7902
JX8R	GTCTTCTTTGGGTCCGTCTGG	9226-9206
JX9R	CAAATACATAGCAATGGGAGTCAAA	10323-10299
JX10R	AACTCGGATGTATGAGGCGTAG	11573-11552
JX11R	AAAGCGGGCATACCGTGTAAT	12774-12754
JX12R	TGTTGTTGTTGCTGGCGTTGA	13803-13783
JX13R	TTTGCTGCTTGCCGTTGTTAT	14826-14806
JX14R	AATTACGGCCGCATGGTTCT	15320-15301

*R: reverse primer

Table S2. Primers sequence for CHsx1401 reverse transcription.

Name	Sequence	Position
SX1R	CACTCTTTCGGGAAGGGTGG	1578-1559
SX2R	CAGTCCTGCGCGGTGCGGGGACAG	2539-2516
SX3R	CAATGATGGCTTGAGCTGAGTAT	2878-2856
SX4R	AGCGACCTCGCTGGGGGCGGTT	4029-4008
SX5R	AACACTCCACCGGAGCCCATGGAGG	5303-5279
SX6R	TTTCGCAAGGAAATCCAAGTCTTC	6456-6433
SX7R	CCAAAGCGTGCCATCGATCCC	7622-7602
SX8R	GTCTTCTTTGGGTCCGTCTGA	8926-8906
SX9R	CAAAGACATAGCAGTGAGAGTCAAA	10023-9999
SX10R	AACTCGGATGTACGATGCATAA	11273-11252
SX11R	AAGGCGGGCACACCGTATAAT	12474-12454
SX12R	TGCTGTGGTTGTTGGCGCTGA	13503-13483
SX13R	TTTGCTGTCTGCCGTTGTTAT	14526-14506
SX14R	AATTTCGGCCCATGGTTCTCGCCAATTAAAT	15021-14990

*R: reverse primer
